# An easy way to retrieve two specimens with one snare

**DOI:** 10.1055/a-2333-9552

**Published:** 2024-06-25

**Authors:** Song Su, Zimeng Wang, Yawei Bi, Longsong Li, Nanjun Wang, Enqiang Linghu, Ningli Chai

**Affiliations:** 1 Department of Gastroenterology, The First Medical Center of Chinese PLA General Hospital, Beijing, China

Endoscopic resection of multiple colonic polyps is very common in clinical practice. Successful retrieval of the specimens enables histopathologic evaluation of polyp tissue, which helps determine complete eradication, subsequent management, and endoscopic surveillance interval 1 2 . However, some specimens may not be extracted by aspiration. In this situation, repeat colonoscope insertion for retrieval of multiple specimens is not only time consuming, but also carries additional risks and discomfort, especially when retrieving polyps in the ascending or transverse colon. Moreover, the process of searching for specimens can at times be extremely frustrating and further prolong the operation time. Herein, we report an easy way to simultaneously retrieve two specimens with one snare, which can reduce the number of repeat colonoscope insertions.

A 67-year-old man with a 2.5-cm laterally spreading tumor ( Fig. 1 **a** ) in the transverse colon, and multiple polyps in the hepatic flexure and ascending colon, was referred to our center. The polyps were 0.5–1.2 cm in diameter, and included a 1.0-cm spherical polyp ( Fig. 1 **b** ) and 1.2-cm flat polyp ( Fig. 1 **c** ). Under sedation, the patient underwent successful endoscopic mucosal resection of the polyps, and all but two specimens (the spherical and flat polyps described above) were retrieved by aspiration. After an hour-long endoscopic submucosal dissection (ESD) procedure for the laterally spreading tumor, and retrieval of the specimen, we reinserted the colonoscope to retrieve the polyp specimens. However, it took a frustratingly long time to find the spherical specimen. Considering the greater risk and discomfort associated with further extending the operation time, as well as the risk that the clips sealing the ESD wound may be dislodged by the colonoscope, we managed to simultaneously retrieve the two specimens with one snare. In brief, we first used the snare to grasp one of the specimens, while the spherical polyp was pulled into the cap. Then, the snare with the specimen was pulled tightly, producing a seal over the cap and preventing the spherical specimen from falling out. Subsequently, with colonoscope withdrawal, the two specimens were successfully retrieved together ( Video 1 ).

**Fig. 1:**
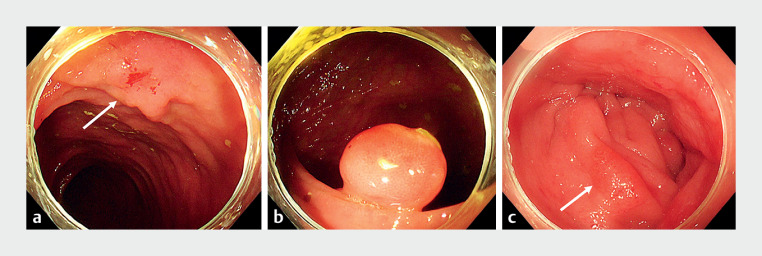
Endoscopy images. **a** Laterally spreading tumor (arrow). **b** Spherical polyp. **c** Flat polyp (arrow).

An easy way to retrieve two specimens with one snare, which helps to reduce the number of repeat colonoscope insertions and associated risks.Video 1

Techniques for simultaneous retrieval of multiple specimens have been scarcely documented. This case demonstrates the usefulness and feasibility of retrieving two specimens simultaneously with a single snare. This very small innovation helps to save time and reduce the risks associated with repeat colonoscopy, and is particularly suitable for lesions in the right colon.

Endoscopy_UCTN_Code_TTT_1AQ_2AC

## Notes

Endoscopy E-Videos https://eref.thieme.de/e-videos
*E-Videos* is an open access online section of the journal *Endoscopy* , reporting on interesting cases and new techniques in gastroenterological endoscopy. All papers include a high-quality video and are published with a Creative Commons CC-BY license. Endoscopy E-Videos qualify for HINARI discounts and waivers and eligibility is automatically checked during the submission process. We grant 100% waivers to articles whose corresponding authors are based in Group A countries and 50% waivers to those who are based in Group B countries as classified by Research4Life (see: https://www.research4life.org/access/eligibility/ ). This section has its own submission website at https://mc.manuscriptcentral.com/e-videos .
